# The mechanism of mammalian peroxidase destruction of invasive microbes

**DOI:** 10.1371/journal.pone.0339356

**Published:** 2026-01-22

**Authors:** Razvan Puf, Michael L. Smith, Aatto Laaksonen

**Affiliations:** 1 Centre of Advanced Research in Bio-nanoconjugates and Biopolymers, Petru Poni Institute of Macromolecular Chemistry, Iasi, Romania; 2 Medical Chemistry, Umeå University, Umeå, Sweden; 3 Department of Chemistry, Stockholm University, Stockholm, Sweden; 4 State Key Laboratory of Materials-Oriented and Chemical Engineering, Nanjing Technical University, Nanjing, China; 5 Department of Engineering Sciences and Mathematics, Luleå University of Technology, Luleå, Sweden; Universidade Federal do Para, BRAZIL

## Abstract

We calculated the internal energies (ΔE) for the breakdowns of HOI, HOBr and HOCl for the first time using the principles of molecular orbital theory. The release of atomic oxygen (ATOX) from all three molecules was estimated being from 43.3 (HOCl) to 64.1 (HOI) kcal mol^−1^. These internal energies are much less than the inputs required for hydroxyl anion and cationic halide productions which range from 315.0 (HOI) to 381.1 (HOCl) kcal mol^−1^. These results answer the puzzle concerning the fates of the products from the halide oxidations by peroxidases. The active species were thought to be the hypohalous acids themselves or the cationic halide but ATOX has never been considered. ATOX is an electron pair accepter and an incredibly destructive species which is observed only in high energy systems. Our results have implications for mammalian immunology because the final steps for microbe disposal in mammals are destructions by one of three peroxidases; lactoperoxidase (LPO), eosinophil peroxidase (EPO) or myeloperoxidase (MPO). These all utilize H_2_O_2_ and one of the halide ions; I^−^ (LPO), Br^−^ (EPO) or Cl^−^ (MPO) to biosynthesize HOI, HOBr and HOCl, respectively. The low energies required for ATOX liberation from hypohalous acids explains why these are the preferred products of important mammalian peroxidases. For example, LPO is an integral enzyme of mammalian airway defence and enhanced nutritional iodine intake encourages liberal biosynthesis of HOI, which is immediately lethal to all microbes tested *in vitro* and *in vivo.*

## Introduction

We calculated the internal energies, ΔE, for the spontaneous disintegrations of the hypohalous acids, HOI, HOBr and HOCl for the first time. Hypohalous acids are inherently unstable, have never been purified and are probably explosive so the disintegration energies were previously unknown. Three peroxidase (PO, EC 1.11.1.7) enzymes which biosynthesize hypohalous acids are known to be intimately involved in human immunity; lactoperoxidase (LPO) in airway mucus, saliva [1], tears and milk, eosinophil peroxidase (EPO) with eosinophils and myeloperoxidase (MPO) located in the granulocytes of neutrophils, macrophage and natural killer (NK) cells. All three are capable of destroying most any bacteria, fungus or virus [2,3]. These require two substrates for hypohaloacid biosyntheses. The first is oxygen donation to the resting PO by H_2_O_2_ and the second reduces the oxygen-PO complex back to the resting PO. For LPO this electron donor can be either I^−^ or SCN^−^ both which can be utilised by all three PO enzymes, Br^−^ by EPO and MPO, Cl^−^ which can only be oxidized by MPO. Some properties of HOCl in biology have been recently reviewed [4]. The topic of reactive oxygen species (ROS) has been recently reviewed though atomic oxygen (ATOX) is not mentioned [5,6].

The two general reactions we examined were the release of atomic ATOX with the related acid halide and production of hydroxyl anion with cationic halide. The example reaction producing I^+^ is summarized as

$$HOI\to HO^{-}+I^{+}.$$ (1)

We suspected the major products from HOI, HOBr and HOCl might be the related simple acids and ATOX with an example as

HOI→HI+O. (2)

This latter reaction was only a suspicion since the former, reaction (1) was previously thought as a possible path of HOI disintegration with important practical applications [7]. The activation of iodine, often radioactive iodine, by LPO and H_2_O_2_ is a historical laboratory tool used for labelling proteins, tyrosyl moieties and membranes [8]. This, and the biosynthesis of T_4_ by both TPO and LPO, are evidence that some iodide ion is oxidized into I^+^.

Reactions (1) and (2) occur far from equilibrium and being irreversible the energetics, for instance the free energies (ΔF), are nearly impossible to measure. ATOX is difficult to detect by any means, it is invisible, displays no magnetic moment and can only be detected using high energy ultraviolet light. This means ATOX spectroscopy is nearly impossible in a living system or tissue models. These difficulties make the study of ATOX and cationic halide productions problematic. Molecular orbital (MO) calculations of the internal energies are the only way to gain insight into these important reactions. We found that the reactions producing ATOX and cationic halides are all endothermic, requiring energy input. We were surprised to find that breakdowns into ATOX, require much less energy than cationic halide productions. This means that ATOX is the primary product from hypohaloacid disintegrations.

These hemeproteins share similar structures with all genes located on human chromosome 17 [3]. The biosyntheses of hypohalous acids catalysed by POs have been investigated in detail. The overall two step reaction, with I^−^ as the halide, is summarized as

$$LPO(Fe^{+3})+H_{2}O_{2}\to Fe^{+5}=O+H_{2}O\;Fe^{+5}=O+I^{-}\to LPO(Fe^{+3})+OI^{-}$$ (3)

where *Fe*^+5^ = *O* represents oxygen bound directly to the hemin iron and *LPO*(*Fe*^+3^) represents LPO in the unoxidised, resting state. Iodide is more rapidly utilized than Br^−^ or Cl^−^ by all three enzymes. The *Fe*^+5^ term is simply a formality with much positive charge delocalized throughout the large porphyrin *π*-electron cloud. Evidence for the biosynthesis of HOI is firm, a recent structural study on crystalline LPO located the HOI product at several sites about the enzyme [9]. This is different chemistry from oxygen activation by cytochrome P450 where the hemin-oxygen complex is the reactive species.

For these reasons we investigated the energetics for the breakdowns of three hypohalous acids, HOI, HOBr and HOCl for the first time. We used molecular modelling with a high speed, large memory computer following the principles of atomic chemistry. We calculated the internal energy differences between the reactants and the two product ensembles for each hypohalous acid. The release of ATOX along with simple halide acids is energetically favoured over the cationic halide and hydroxide productions. We found the former pair, reaction 1, though previously believed to be important products, are only minor products. There is nothing subtle about ATOX, it is an incredibly reactive nuisance which has only heretofore been observed in nature as a product in high energy physics [10]. We now understand why peroxidases, key players of the mammalian immune system, catalyse productions of hypohalous acids.

## Methods

All calculations were performed as High Performance Computing *via* the National Academic Infrastructure for Supercomputing in Sweden (NAISS) on the Tetralith supercomputer at Linköping University. The description of this device can be found at https://www.naiss.se/resource/tetralith/. The Density Functional Theory (DFT) method was used to determine the energies of both the reactants and products [11]. For this we relied on the B3LYP hybrid functionals with the LANL2DZ basis set [12], as well as the M06-2X functional [13] with the aug-cc-pVDZ basis set [14]. For the iodine atom, the aug-cc-pVDZ-PP basis set was employed to account for relativistic effects at work within this electron rich atom [15]. The halogen atom parameters used with LANL2DZ, as well as those for the aug-cc-pVDZ and aug-cc-pVDZ-PP basis sets, were extracted from the Basis Set Exchange website [16]. Calculations were performed using the Gaussian 16 software package [17]. The calculations times were quite lengthy and demanding due to inclusion of Br and especially for I compounds.

We calculated the internal energies for the spontaneous release of ATOX from hypohalous acids using first principles with density-functional theory (DFT). We also calculated the energies for release of the cationic halides, I^+^, Br^+^ and Cl^+^, with simultaneous production of HO^−^ which is the other likely path of disintegration. The energies, relative to the hypohalous acids, were calculated stepwise as functions of nuclear separation distances during product formation. The interatomic distance data can be found at https://github/mlsmith55/MolecularDistances in .xyz format.

The energies required for these bond breakages are estimated with the general relationship

$$\Delta E=E_{d}-\Delta H_{f}$$ (4)

where *E*_*d*_ is the energy of dissociation and the enthalpy of formation the chemical species is $\Delta H_{f}$. Since the medium for hypohaloacid disintegration in biology is aqueous and isothermal, the pressure-volume effects are small and were neglected. Allowing this approximation we neglect the small differences expected between the internal energies and enthalpies.

For calculating the energies associated with ATOX release we use the relationships below where O  represents ATOX

$$\Delta E_{O^{*}}=E_{d}^{HOX}-(\Delta H_{f}^{HX}-\Delta H_{f}^{O^{*}}).$$ (5)

Here $\Delta E_{O^{*}}$ is the internal energy of ATOX, $E_{d}^{HOX}$ the disintegration energy of hypohaloacid, $\Delta H_{f}^{HX}$ the enthalpy of halide acid formation and $\Delta H_{f}^{O^{*}}$ the enthalpy of formation of ATOX. For calculating the energies associated with the spontaneous cationic halide release we use the relationships below where *X*^+^ represents the cationic halide

$$\Delta E_{X^{+}}=E_{d}^{HOX}-(\Delta H_{f}^{HO^{-}}+IE^{X^{+}}).$$ (6)

Here $\Delta E_{X^{+}}$ is the internal energy of the cationic halide, $E_{d}^{HOX}$ the disintegration energy of the hypohaloacid, $\Delta H_{f}^{HO^{-}}$ the enthalpy of formation of OH^−^ and $IE^{X^{+}}$ the ionization energy of the halide.

## Results

The Hartree values calculated for the individual species after geometry optimization are listed in Table 1. The trend of increasing energy correlating with decreasing halogen atomic number is observed for all three forms; hypohaloacid, halide acid and cationic halide. Also expected is the small energy difference between the hydroxide anion and the oxygen atom. These values were used to calculate the internal energies for the species listed in Table 2.

**Table 1:** Hartree energies obtained from DFT calculations using B3LYP functionals with the LANL2DZ basis sets for reactants and products. The energy estimates were made without the presence of solvent (water).

Species	Energy (Hartree)
HOI	-87.2
HI	-12.0
I^+^	-11.0
HOBr	-88.9
HBr	-13.8
Br^+^	-12.6
HOCl	-90.7
HCl	-15.5
Cl^+^	-14.3
HO^−^	-75.7
O	-75.1

**Table 2:** Internal energies for the disintegration of all three hypohalous acids calculated from the Hartree energies of Table 1 (B3LYP functionals).

Reactants	Products	ΔE (kcal mol^−1^)
HOI	HI + O	64.1
HOI	HO^−^ + I^+^	315.0
HOBr	HBr + O	53.9
HOBr	HO^−^ + Br^+^	345.5
HOCl	HCl + O	43.3
HOCl	HO^−^ + Cl^+^	381.1

**Fig 1:**
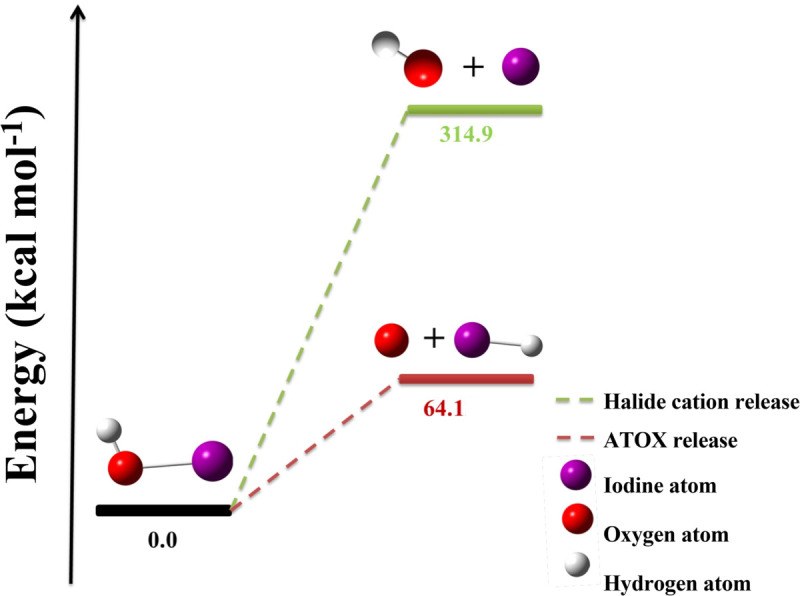
Internal energies (ΔE) for the disintegration of HOI.

The important results are the large differences between the energies of ATOX production and those of cationic halide with hydroxide productions are listed in Table 2) and presented in Fig 1 for disintegration from HOI. The average difference is a whopping 293 kcal mol^−1^, close to that calculated between the two paths for HOBr disintegration of 292 kcal mol^−1^. Note the expected correlation of decreasing energy input required to create the cationic halides with decreasing halide electronegativity. That is, the creation of the ion pair requires less energy input from HOI than from HOCl, which is reassuring. Also, the release of ATOX from HOI is less favourable than release from HOBr, which is less favourable than from HOCl, again correlating with halide electronegativity. The important finding is the great difference between disintegration energies with path, ATOX being very much preferred rather than cationic halide. This is in large part due to the energy required for halide ionization along with electrical charge separation. The large energy inputs being necessary to form the cationic halides are probably dampened, but only slightly, in the aqueous environment by charge delocalisation of both cation and anion [18].

The ground state, left, the products ATOX and HI or HO- and I+, middle and top. Both product ensembles require significant energy input; much more energy required for producing the HO- and I+ pair than ATOX with the acid halide.

The internal bond strength of only hypobromous acid has been previously reported. The strength of the O-Br bond of the HOBr molecule was determined from the electronic spectrum in the UV region [19]. The value of 48.5 ±0.4 kcal mol^−1^ is in the neighbourhood of our 53.9 kcal mol^−1^ though these are estimates of different but related bonds. This value is evidence, however, that our bond energy estimates are close to reality.

We also calculated the internal energies for disintegrations using the M06-2X functionals with the aug-cc-pVDZ basis sets. The energetics for ATOX release are listed in Table 3. Note the trend of requiring less energy input following the halogen electronegativity holds for these calculations, too. The differences between these results, which average about 13.9 kcal mol^−1^, give us an idea of the probable systematic errors in our calculations.

**Table 3:** Internal energies for the disintegration of three hypohalous acids calculated from the M06-2X functionals. The energy estimates were made without the presence of solvent (water).

Reactants	Products	ΔE (kcal mol^−1^)
HOI	HI + O	80.2
HOBr	HBr + O	65.7
HOCl	HCl + O	57.0

## Discussion

Using molecular orbital theory we investigated the energetics for the disintegrations of the hypohalous acids, HOI, HOBr and HOCl. Our results demonstrate the spontaneous release of ATOX from these is greatly favoured over cationic halide with OH^−^ formations. One reason for the large energy difference is the energies required for electron transfer from the halide to OH along with charge separation. While it is difficult to calculate the reaction free energies at this time, we know that the halide acids are stable in aqueous solution and the associated dissolution free energies will be negative. Because of these reasons we think the reaction free energies in water will follow our calculated trends. We hope to improve our calculations with more detailed investigations and present the reaction free energies in water soon.

The fundamental physics and short lifetime of ATOX makes this species very difficult to detect in the laboratory. With four electron pairs tightly held by eight protons and eight neutrons, ^16^O has no magnetic moment and suffers no electronic transition at energies less than high frequency ultraviolet radiation. This means that ATOX might only be detected using short burst, laser radiation. Another spectral region which may be informative is the microwave, which might be able to excite unique rotational transitions though these would likely be broadened in water. Since ATOX is very reactive, more experimental evidence following ATOX reactivity should be rather easily uncovered. The excited triplet state, O(3P), has been prepared in model systems and it preferentially oxidizes primary thiols such as cysteine, also aryl and alkene hydrocarbons [20,21]. The only oxygen species with an interesting magnetic signal is the expensive ^17^O. This could be incorporated into H_2_O_2_ by either glucose oxidase or a duox enzyme and after activation by a PO the products detected by NMR. The magnetic properties of ^17^O will be highly dependent on neighbouring atoms and readily diagnostic. Both nearby ^13^C and ^1^H magnetic signals will be mightily affected by a neighbouring ^17^O.

There are several experimental observations consistent with our results. A target protein myoglobin was examined by mass spectroscopy after treatment with HOCl, modelling MPO action. The MW increased by steps of 16 a.m.u. but very little by 35 and 37 a.m.u. (Cl), consistent with ATOX addition [22]. In another experiment there were indications of both ATOX and I^+^ activities when bovine and mouse serum albumins were targeted in a system utilizing MPO with iodide substrate [23]. In a third study, the metalloproteinase, human matrilysin (MMP-7), was exposed to the action of MPO and the MMP-7 lost four a.m.u. [24]. This is simply explained as two ATOXs extracting four H· becoming two waters. Our results are also consistent with the power of the LPO system to liquidate all microbes tested *in vitro* [2,25].

Many organisms biosynthesize H_2_O_2_, a strong oxidizing agent and deadly to many microbes. To counter H_2_O_2_ some bacteria, *M. tuberculosis* for instance, synthesize catalase-peroxidase (CP) enzymes which rapidly disproportionates H_2_O_2_ into the relatively innocuous dioxygen and two waters [26]. CP performs this without any direct energy input from the bacterium. To circumvent this economical defence, mammals have evolved peroxidases producing hypohalous acids and ATOX.

The smaller energy input required to create ATOX rather than an ion pair, means this reaction is greatly preferred. This also means that ATOX production should be significantly enhanced when the temperature is raised by only a few degrees, for instance, during mammalian fever. This answers the very old question of why endotherms develop fever when infected. The *how* is primarily the activity of the mitochondrial uncoupling protein generating heat rather than ATP. One might think microbes to be thermally unstable near 37°C as the reason for fever, but bacterial and viral agent instability with such a small T increase is unlikely. Instead, we think a small temperature increase stimulates hypohalous acid biosyntheses by EPO and especially MPO. Because the activation energy of an endothermic reaction is usually close to the overall required energy, the disintegration of HOCl into ATOX and HCl, not requiring enormous energy input, will be enhanced by fever.

Because ATOX oxidation cannot be controlled, the activities of mammalian POs are always segregated from healthy tissues. Lactoperoxidase never comes in contact with host cells, EPO and MPO only to eliminate dangerous microbes after careful activation. The results of MPO activities are simultaneous microbe destruction along with the phagocyte liquidation and nearby host cells are often damaged in the process. Two other POs likely generate ATOX, thyroid peroxidase, requiring I, and peroxidasin, utilizing Br to cross-link extracellular collagen. Both are activities are partitioned from most living tissues, obviously necessary to avoid uncontrolled tissue damage and inflammation [27,28]. We hypothesize that to avoid random tissue destruction, mammals have developed a set of halide-dependent peroxidases, absent from most cell types, producing hypohalous acids but only when and where necessary.

The nutritional intake requirement for Br is currently unknown and the human biochemistry is poorly studied. The chief source of bromine is likely table salt. While bromine is required by eosinophil activity Br is also a mutagen if bromouracil is biosynthesized by "mistake". This situation is complicated, Br is required for defence and collagen biosynthesis but it also modifies DNA causing mutations [29]. The biochemistry and cell biology of bromine needs much more investigation.

The biosynthesis of hypohalous acids can be substrate limited. This is not a problem for the synthesis of HOCl by MPO where Cl^−^ is plentiful but the concentrations of Br^−^ and I^−^ can be limiting [25]. It is estimated that over 2 billion people are iodine deficient [30]. Maintaining proper iodine concentration is paramount for good health but often difficult because nutritional sources are rare. Iodine is very sparse in plant foods, sea salt and highly variable in animal sources, even fish. By far the best sources of iodine are seaweed and kelp which are only commonly eaten in Japanese and some Korean cultures. Certain processes around iodine biochemistry involved with hormone biosynthesises are understood but iodine transport into the leukocytes and mucus needs investigation, especially considering the influence of industrial pollutants [31].

**Fig 2:**
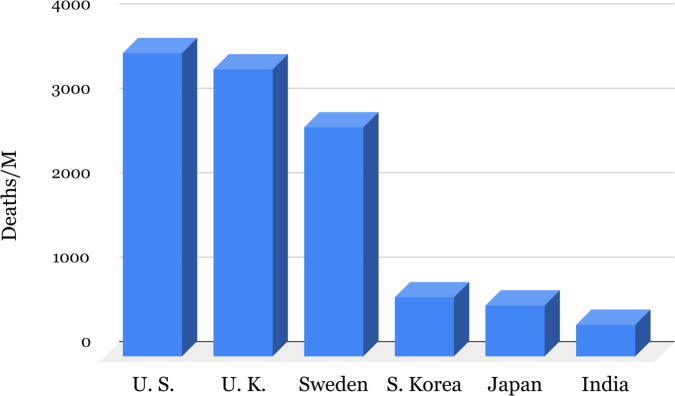
Deaths per million inhabitants due to COVID-19, 2019 until September, 2025.

The results of our calculations for ATOX production helps explain the mechanism for the selective resistance to the recent COVID-19 pandemic. Fig 2 presents some national death numbers during the COVID-19 pandemic, crowded Japan reporting puny numbers compared to the U.S., U.K. and Sweden. The likely reason for this outcome is the Japanese diet which is iodine rich, encouraging liberal HOI production, therefore ATOX, by LPO in human airways [32]. Since ATOX generated by LPO is a general biocidal, the protective effect from the liberal use of nutritional iodine will provide protection against many other microbes. A recent clinical study reported that povidone-iodine (PVD-I) applied to the nasal region immediately eradicates the SARS-CoV-2 virus in human airways [33] and PVD-I application reduces SARS-CoV-2 infections [34]. The ability of PVD-I to destroy airborne viral agents has been known and widely used, for many decades, by Indians [35] data from the WHO COVID dashboard September 2025 [36].

## Conclusions

The internal energy differences, ΔE, between the products, ATOX *vs.* cationic halide, I^+^, Br^+^ and Cl^+^ from the respective hypohalous acid disintegrations, are all very large, greatly favouring ATOX. Incredibly active ATOX is the major product from hypohalous acid breakdowns after biosyntheses, catalysed by mammalian peroxidases, not cationic halides.
